# Nisin-like biosynthetic gene clusters are widely distributed across microbiomes

**DOI:** 10.1128/mbio.01545-25

**Published:** 2025-09-10

**Authors:** David Hourigan, Des Field, Ellen Murray, Ivan Sugrue, Paula M. O’Connor, Colin Hill, R. Paul Ross

**Affiliations:** 1 APC Microbiome Ireland, Biosciences Institute, Biosciences Research Institute, University College 8795, Cork, Ireland; 2 School of Microbiology, University College Cork, University College 8795, Cork, Ireland; 3 Teagasc Food Research Centre Moorepark 34344 , Fermoy, Co. Cork, Ireland; McMaster University, Hamilton, Ontario, Canada

**Keywords:** bacteriocins, natural antimicrobial products, mobile genetic elements, antimicrobial peptides, heterologous gene expression, microbiome, genomics

## Abstract

Bacteriocins are antimicrobial peptides/proteins that can have narrow or broad inhibitory spectra and remarkable potency against clinically relevant pathogens. One such bacteriocin that is extensively used in the food industry and with potential for biotherapeutic application is the post-translationally modified peptide, nisin. Recent studies have shown the impact of nisin on the gastrointestinal microbiome, but relatively little is known of how abundant nisin production is in nature, the breadth of existing variants, and their antimicrobial potency. Whether or not nisin production and immunity are widespread in gut microbiomes could be a deciding factor in determining the suitability of nisin as a prospective therapeutic for human and/or animal infections. Here, we used publicly available data sets to determine the presence of widespread and diverse nisin biosynthetic gene clusters (nBGCs) across the biosphere. We show that 30% of these nBGCs are predicted to be located on mobile genetic elements, with some found in pathogenic bacteria. Furthermore, we highlight evidence of horizontal gene transfer of nBGCs between genera, including *Streptococcus suis*, *Enterococcus hirae*, and *Staphylococcus aureus*. In all, we describe 107 novel nisin-like peptides. Five representatives were heterologously expressed and all exhibited antimicrobial activity. We further characterized nisin VP, a novel natural nisin variant produced by *Velocimicrobium porci* isolated from the porcine gut. The peptide has a completely novel hinge region “AIQ” not detected in other nisin variants to date. While nisin VP could be induced by nisin A, the latter could not be induced by nisin VP.

**IMPORTANCE:**

Our research reveals the heretofore underappreciated presence of diverse and widespread nisin-like biosynthetic gene clusters in microbiomes across the globe. Notably, different clusters share similar biosynthetic machinery but differ in sequence, suggesting gene transfer and adaptation. We identify >100 new nisin-like variants, including several in species not previously known to produce nisin. This emphasizes the widespread dissemination of nisin-like gene clusters and the diversity of novel core peptides with biotherapeutic potential. These findings point to a role for nisin in microbial competition in microbiomes. We heterologously expressed nine nisin variants, five of which are completely novel peptides, using the nisin A biosynthetic machinery and confirmed that all exhibited antimicrobial activity.

## INTRODUCTION

Bacteria are in constant competition for nutrients in different environmental niches. A favorable trait that supports colonization and survival in a competitive niche is the production of antimicrobials that could provide an advantage over competitors (1, 2). Bacteriocins are a group of ribosomally synthesized antimicrobial peptides/proteins produced by bacteria with either both broad or narrow host ranges (3). Due to their potency and efficacy against clinically relevant bacteria, they are often touted as a potential natural alternative to traditional antibiotics. The bacteriocin nisin was first discovered by Rogers almost a century ago and, since then, has become one of the most extensively studied antimicrobial peptides (4). First identified in *Lactococcus lactis*, it has potent antimicrobial activity against many Gram-positive strains and has gained approval by the Food and Drug Administration (FDA) and the European Food Safety Authority (EFSA) as a food preservative (5). To date, there have been 15 characterized nisin variants across a number of different genera, including *Streptococcus*, *Staphylococcus*, *Romboutsia*, and *Blautia* (6–12). Research over the past decade has highlighted the biotherapeutic potential of nisin as a natural alternative to antibiotics due to its broad spectrum of inhibition for Gram-positive pathogens, including methicillin-resistant *Staphylococcus aureus* (MRSA), vancomycin-resistant *Enterococcus* (VRE), *Streptococcus pneumoniae*, *Bacillus cereus*, and *Listeria monocytogenes* (8, 13, 14). Nisin has also had efficacy against bacteria associated with mastitis, skin, and soft tissue infections, as well as pathobionts such as *Cutibacterium acnes* and *Clostridioides difficile*, associated with acne vulgaris and recurrent *C. difficile* infection (rCDI), respectively (8, 15, 16). However, several challenges hinder nisin’s therapeutic use, including its poor solubility, limited stability at physiological pH, resistance mechanisms in target bacteria, and susceptibility to proteolytic digestion in the upper gastrointestinal tract (17–21). Natural nisin variants have activity against gut commensals and pathogens such as Lan-Df, a nisin variant identified in *Dorea formicigenerans* (16). This peptide has potent activity against the gut commensal *Blautia obeum* (0.01 µM) and even greater activity than nisin A (<1 µM) against *C. difficile* VPI10463 (16, 22).

Recent studies highlight the biotherapeutic potential of nisin, suggesting that nisin could be one of the first bacteriocins approved for clinical use (23). However, only limited applications of the use of nisin in model clinical settings have been described (16, 24–27). Notably, purified nisin selectively depleted *C. difficile* in an *ex vivo* colon model (23, 28). It has been known since the 1990s that the operons for nisin A and Z production are localized on conjugative transposons, including Tn5276 and Tn5278 from *L. lactis* NIZO R5 and *L. lactis* ILC11, respectively (29, 30). Since then, multiple nisin-like BGCs (nBGCs) have been identified on mobile genetic elements (MGEs). Nisin H from *Streptococcus hyointestinalis* DPC6484 is co-localized with two transposases, while suicin from *Streptococcus suis* 90-1330 is located on an integrative and conjugative element (ICE), and nisin J is located on the plasmid pJOS01 (6, 8, 9). An interesting note is that pJOS1 lacks the nisin regulatory elements and contains a putative transposase, suggesting the cointegration of MGEs (8). The presence of multiple nBGCs on separate MGEs suggests further widespread dissemination (8, 29, 31).

The nisin dehydratase (LanB) and nisin cyclase (LanC) machinery responsible for the dehydration/elimination and cyclization steps, respectively, are required for peptide maturation (32). These modifications give nisin the five characteristic rings that are critical to its antimicrobial activity. Rings A and B are responsible for binding lipid II and contain a conserved “CTPG” region (33). The full nisin A BGC includes genes encoding NisA (prepropeptide), NisB (dehydration/elimination), NisC (cyclization), NisFEG (immunity pumps), NisT (transporter), NisP (prepropeptide cleavage), NisRK (regulation system), and NisI (dedicated immunity protein) (34). Most natural nisin variants share these genes but have varying degrees of identity (and order). This is the case for LanC and LanP for nisin P that share 92.6% and 36.1% amino acid identity with the machinery responsible for nisin A production in *L. lactis* (35). This variance is also mirrored in the immunity proteins (7). These varying degrees of amino acid identity suggest “domestication” after horizontal gene transfer (HGT). With growing genomic databases and evidence of nBGCs on MGEs, we searched the nr and MGnify databases for novel nisin variants. We report 164 unique core peptides, of which 107 are novel nisin-like peptides when accounting for sequences previously reported in other *in silico* screens (36). We show that 30.7% of these are predicted to be located on MGEs. We also show that some nBGCs are found in bacteria previously unknown to produce nisin, such as *Velocimicrobium porci* and *Tsukamurella paurometabola*. Nine of these peptides were subsequently heterologously expressed in *L. lactis* MG1614, and all displayed antimicrobial activity. Five of these are novel nisin variants, including nisin VP produced by the anaerobic pig gut isolate *Velocimicrobium porci*.

## RESULTS

### Widespread distribution of Nisin-like biosynthetic gene clusters

**Fig 1:**
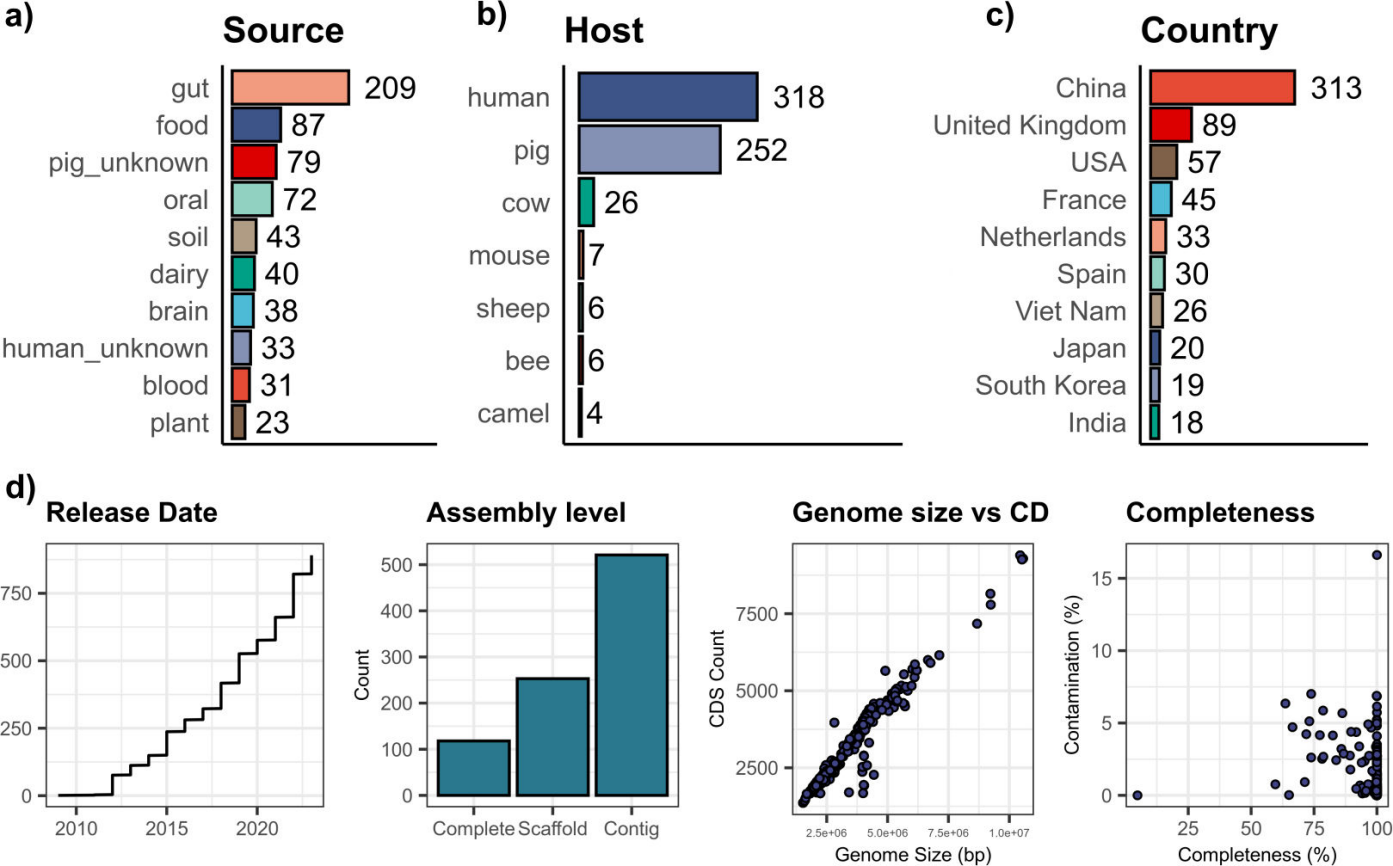
Nisin-like biosynthetic gene clusters are widespread, abundant, and diversely distributed. (**a**) Gut and food isolation sources represent the largest known sources. (**b**) Human and pig hosts are the largest source of nisin-like BGCs. (**c**) China, the United States, and the United Kingdom represent the countries with the most nBGCs. (**d**) Genome assembly metadata for 915 genomes in this study.

We used LanB as a driver sequence to initiate our search. A total of 19,406 LanB hits were identified in the National Center for Biotechnology Information’s (NCBI) nr database, based on a search encompassing 567,228 bacterial genomes. Hits below an *E*-value of 1e-05 across the whole protein were retained. Rodeo was used to retrieve the genomic context of 14,758 putative nisin nBGCs. The predicted core peptides were manually curated based on the core peptide sequence (37). This reduced the final number of putative nisin-like BGCs to 888. The MGnify databases included 27 representative-level genomes with nBGCs, which resulted in a total of 915 nBGC-containing genomes. On average, approximately 60 genomes encoding a nBGC are uploaded to NCBI every year. This increased to 94.8 when data were taken over the last five years exclusively. The majority of nBGCs were uploaded from China, the United States, the United Kingdom, and the Netherlands (Fig. 1c), almost certainly reflecting the fact that these countries sequence and deposit to NCBI more frequently. Similarly, the metadata quality varied between accessions (Fig. 1d). Therefore, the metadata was manually curated to refine the data set. A total of 49 entries (5.4%) had missing data for both source and host.

The principal known isolation source of bacterial genomes was the gut and oral microbiota, with 23.9% (209/915) and 7.9% (72/915), respectively (Fig. 1a). These accessions included *Blautia*, *Ruminococcus*, *Dorea*, and *Velocimicrobium*. Of note were the number of isolates from brain abscesses and blood sources that were dominated by *S. suis* species from both pig and human hosts (6.89%, 63/915). Although nBGCs were found in bacteria from both human and animal clinical isolates, this does not infer that these gene clusters played a role in virulence. Interestingly, nBGCs were also found in an uncut heroin sample encoded in the spore former *Bacillus subtilis* 7702 and in *Bifidobacterium* sp. ESL0798 isolated from a Brazilian bee gut microbiota. The main hosts were humans (34.8%, 318/915), pigs (27.5%, 252/915), and cows (28.4%, 26/915) (Fig. 1b; Table S1). This aligns with evidence of recently discovered natural nisin variants nisin E, suicin, and nisin J from cow, pig, and human sources, respectively (7, 8, 11). This also agrees with recent evidence of nBGCs found in bacteria from gut origins (16).

### nBGCs are widespread and biosynthetic machinery has a highly conserved structure

**Fig 2:**
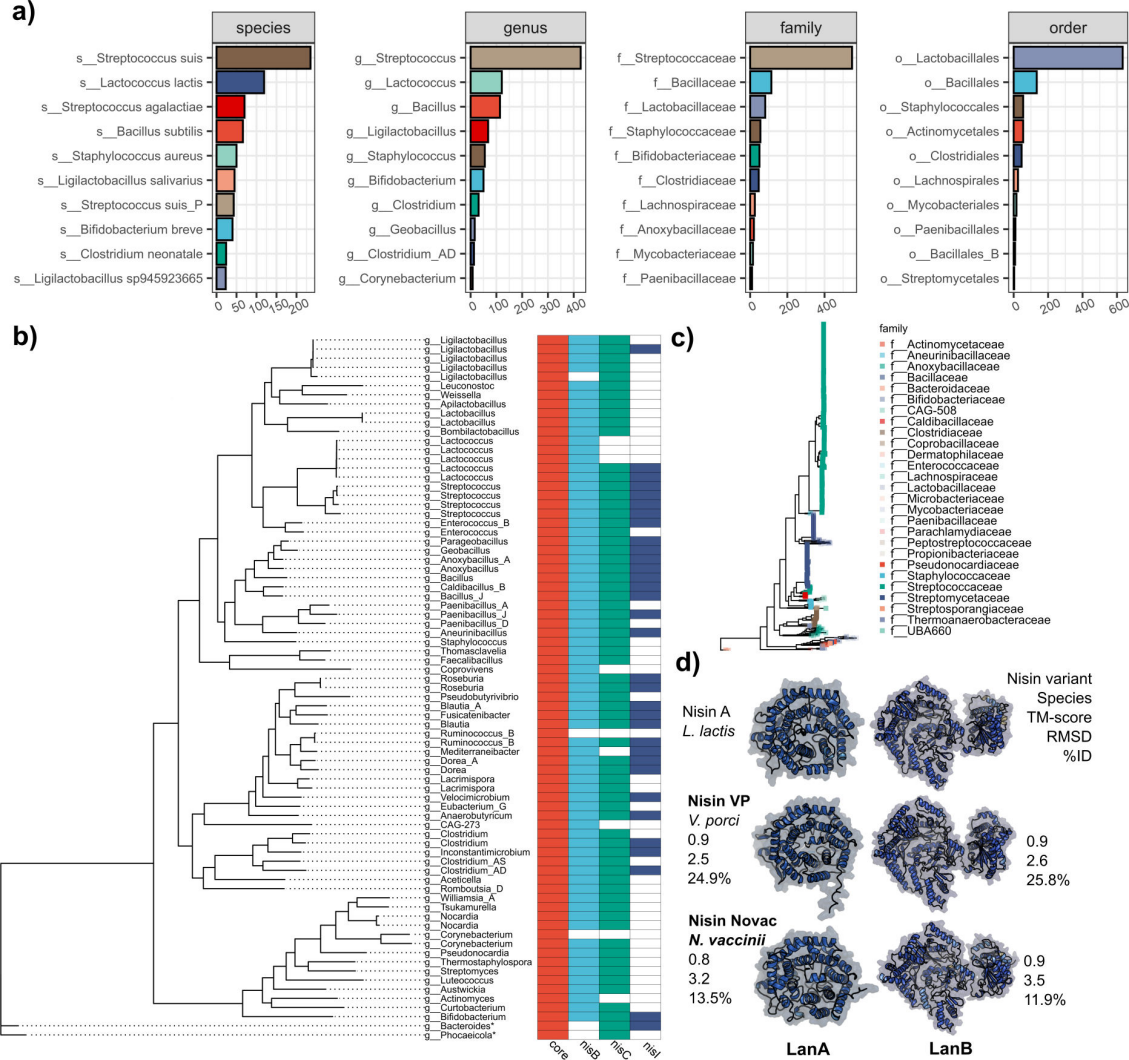
Nisin-like biosynthetic gene clusters are widespread among Bacillota. (**a**) Overall counts of genomes with nBGCs at the species, genus, family, and order levels. (**b**) A phylogenetic tree showing a subset of the genomes that contain nBGCs and the presence/absence of the LanB, LanC, and LanI proteins. These were chosen based on the diversity of genera and the presence of the nBGC found in MGNIFY. (**c**) A phylogenetic tree of all 915 genomes used in this study. (**d**) Structure prediction for LanB and LanC proteins with AlphaFold2. The protein tertiary structure is colored by confidence in prediction.

To gain insight into the taxonomic distribution of nBGCs, GTDB-Tk was used to apply standardized taxonomy (38). The majority of genomes belong to *Bacillaceae* and *Streptococcaceae*, the families with the most described natural nisin variants to date (8, 11). Two hundred and ninety-four nBGCs were identified among *S. suis* genomes, and 80.6% (237/294) of these were from a porcine host which is known to produce suicin (Fig. 2a) (11). To date, there is a lack of characterized natural nisin variants from the phylum Actinomycetota, which makes up 3.8% of data, with *Bifidobacterium breve* the highest contributor (Fig. 2a). This phylum has previously been shown to encode numerous lanthipeptide BGCs (36). Unusually, a single nBGC was found in a novel *Bacteroides* sp. and in *Phocaeicola dorei*, both of which are Gram-negative organisms. There has been no characterized nisin variant reported for any Gram-negative bacterium; however, evidence is mounting of the presence of lanthipeptide BGCs in their genomes (39). The MGnify database included putative nBGCs encoded in species not known to produce nisin variants, including *Bombilactobacillus mellis* (MGYG000321541) from the honey bee gut, *Anaerobutyricum hallii* (MGYG000000262) from the human gut, and an uncultured novel class of clostridia (MGYG000315312) from a chicken gut microbiome.

In total, nisin peptides were found across 5 phyla, 25 families, 59 genera, and 123 bacterial species. This includes 40 genera that have not been described as nisin-producing, such as *Velocimicrobium*, *Mediterraneibacter* (*Ruminococcus*), *Faecalibacillus*, *Weissella*, and *Anaerobutyricum*. A nBGC cluster was identified on the putative prophage *Streptococcus* phi-SsuZKB4_rum of porcine origin. The contig is 110,533 bp in length, encodes 113 coding sequences, and is an unclassified *Caudoviricetes*. The genome was uploaded to GenBank without an isolation source, and taxonomy could not be assigned using taxmyPHAGE (data not shown). However, further investigation predicted the prophage region on the contig to be 66,573 bp in length with the nBGC localized within this window. Despite this, the region does not appear to represent a complete lysogenic phage (88.2% complete). Rather, the surrounding non-phage gene content is more consistent with remnants of a cryptic phage integrated into a plasmid-like element. It is possible that the BGC may be carried on a MGE resembling a “prophage-plasmid” (40). However, we will refer to this BGC as localized within a predicted prophage region.

We wanted to gain insight into the conservation of the LanBCI machinery. Models for the proteins NisT, NisFEG system, and NisP were initially included in the search but were later omitted due to domains shared across many protein classes within each genome. Overall, 80.0% of 915 genomes contained genes encoding LanB, LanC, and LanI proteins. It should be noted that not all nBGCs contained genes *lanB* and *lanC*. This is the case for a single *Corynebacterium* sp. and a single *Ruminococcus gnavus* assembly, both of which contain orphan nisin core genes (Fig. 2b). An orphan nisin core gene was defined as one not having an associated gene encoding NisB. Although these genes are likely non-functional, they have been retained as part of the core peptide data set. Family-level distribution of the 915 genomes in this study highlights the diversity of organisms with biosynthetic potential (Fig. 2c). We compiled all non-redundant LanB, LanC, and LanI proteins and calculated pairwise alignments for each group (Fig. S1). Three LanC and LanB proteins from *Velocimicrobium porci*, *Nocardia vaccinii*, and *L. lactis* were chosen and compared at the amino acid and 3D structure level based on their low percentage amino acid identity to the canonical *L. lactis* proteins (13.5%–24.9% ID). However, very high structural homology was maintained (TM score >0.9 and RMSD 2.6–3.5) (Fig. 2d). It was also observed that LanB_vp_ from *V. porci* shares key residues in the catalytic domains with LanB_nisinA_, such as Arg83 and Arg14 in the N-terminus region (Fig. S3).

### Nisin-like core peptides are diverse and shared among genera

**Fig 3:**
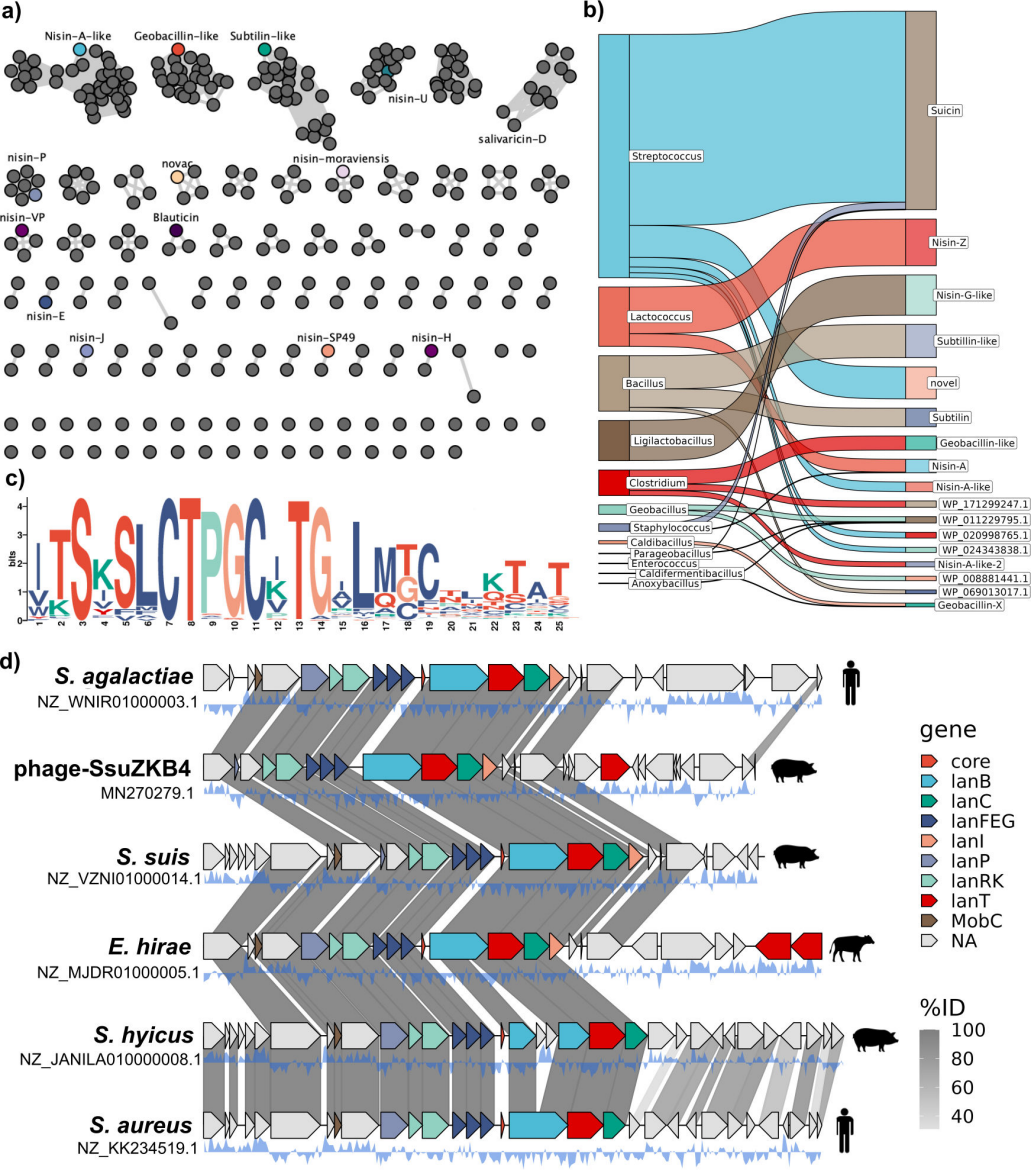
Nisin core peptides have varying degrees of amino acid identity, share key motifs, and are encoded in different genera. (**a**) Sequence similarity network of nisin-like prepropeptides created using EFI-EST. The network contains 314 unique core peptide sequences. This network also contains multiple sequences as predicted by Rodeo. Known natural nisin variants and nisin VP are highlighted. (**b**) A Sankey plot depicting the presence of multiple core peptides within the same genera. Also shown is the presence of the same core peptides shared among different genera. Only counts of the same core sequence above ten were used for the plot. (**c**) A sequence logo for the most conserved motifs among nisin-like core peptides (*n* = 164). The C-terminus end of the core peptide is the most variable. Between the ring structures, positions 4, 12, and 15 were the most variable. (**d**) A synteny plot of six nBGCs that share the same core peptide and leader at 100% amino acid identity. The nBGCs are predicted to be on ICEs. One of these lies in the genome of a phage, phage-SsiZKB4.

In total, 1,060 genes encoding putative core peptides were identified across 915 genomes. We reduced this to 148 unique prepropeptides at 100% amino acid identity identified. The sequence of each core peptide, the originating assembly, contig accession, and taxonomy can be found in Table S2. A table of experimentally verified nisin-like peptides is found in Table S4. Core peptides were shared across genera, suggesting HGT of BGCs across genus-level boundaries (Fig. 3b). We then removed leader sequences and included core peptides identified by Rodeo and missed by Bakta (Table S3) and core peptides with only a protein accession, such as the synthetic variant NisA-AAA (AHA92024.1). This resulted in 164 distinct core peptides (Fig. 3a; File S7). Cleavage sites were determined using canonical “GASPR” and “PQ” cut sites, as observed in nisin and subtilin (41). Where cut sites were deemed too dissimilar, peptides were aligned to their most similar characterized core peptide, and a putative cleavage site was assigned, such as nisin-O and its “PK” cut site (12). It is important to note here the need for a universal naming convention for nisin-like core peptides. Nisin J produced by *S. capitis* APC 2923 shares the same percentage identity with subtilin from *Bacillus subtilis* ATCC 6633 and nisin O from *Blautia obeum* A2-162 (62.9%). As the rate at which variants of this class of bacteriocin are being discovered is steadily increasing, a standardized nomenclature would be useful for future naming of nisin variants, but this is beyond the scope of this study. The most conserved region among the nisin-like core peptides is the “CTPGC” lipid II binding domain, although “CTAGC” motifs were also observed (Fig. 3c). The GTAGC motif resembles the lipid II binding motif of rombocin, a natural nisin variant from *Romboutsia sedimentorum* which lacks ring E (10). Across all unique core peptides, only five amino acids are located at position one, with tryptophan (subtilin-like), isoleucine, or valine being the most highly conserved. The presence of another aromatic residue at position one, tyrosine, is notable, as position one variants of nisin containing an aromatic residue have been shown to be more active against nisin immunity (NisI, NisFEG) and/or nisin resistance determinants, including the nisin resistance protein (Nsr) (42). Core peptide sequences were more conserved than modification enzymes, likely due to their shorter length and essential residues for ring formation (Fig. S1).

We observed that the same prepropeptide (WP_001058290.1) was found in five different species across three different genera *Staphylococcus aureus* (GCF_000644315.1), *Staphylococcus hyicus* (GCF_024580395.1), *Enterococcus hirae* (GCF_002077325.1), *Streptococcus agalactiae* (GCF_009771385.1), and *S. suis* (GCF_018424365.1). These five isolates were isolated from porcine, human, and bovine sources. *S. agalactiae* was isolated from human blood in 2017 (SAMN13340655), and *S. aureus* is MRSA ST398 of human origin (SAMN02404212). The same core peptide was also identified in a putative prophage, *Streptococcus* phage SsuZKB4_rum. To investigate the synteny between nBGCs encoding these identical core peptides, we compared the surrounding 30 kb region at the protein level (Fig. 3d). The *lanB* gene present in S. *hyicus* was found to be interrupted by a gene insertion. However, the remaining nBGCs all have *lanFEGPBCT* determinants. *S. hyicus* and *S. aureus* also lack *lanI* genes, and another gene interruption can be seen in *lanP* in the prophage-SsuZKB4 genome. A gene encoding a MobC protein is also located upstream from LanP across all nBGCs. This protein promotes conjugal mobilization of plasmids and is also found in ICEs (43).

### nBGCs are frequently found on mobile genetic elements

**Fig 4:**
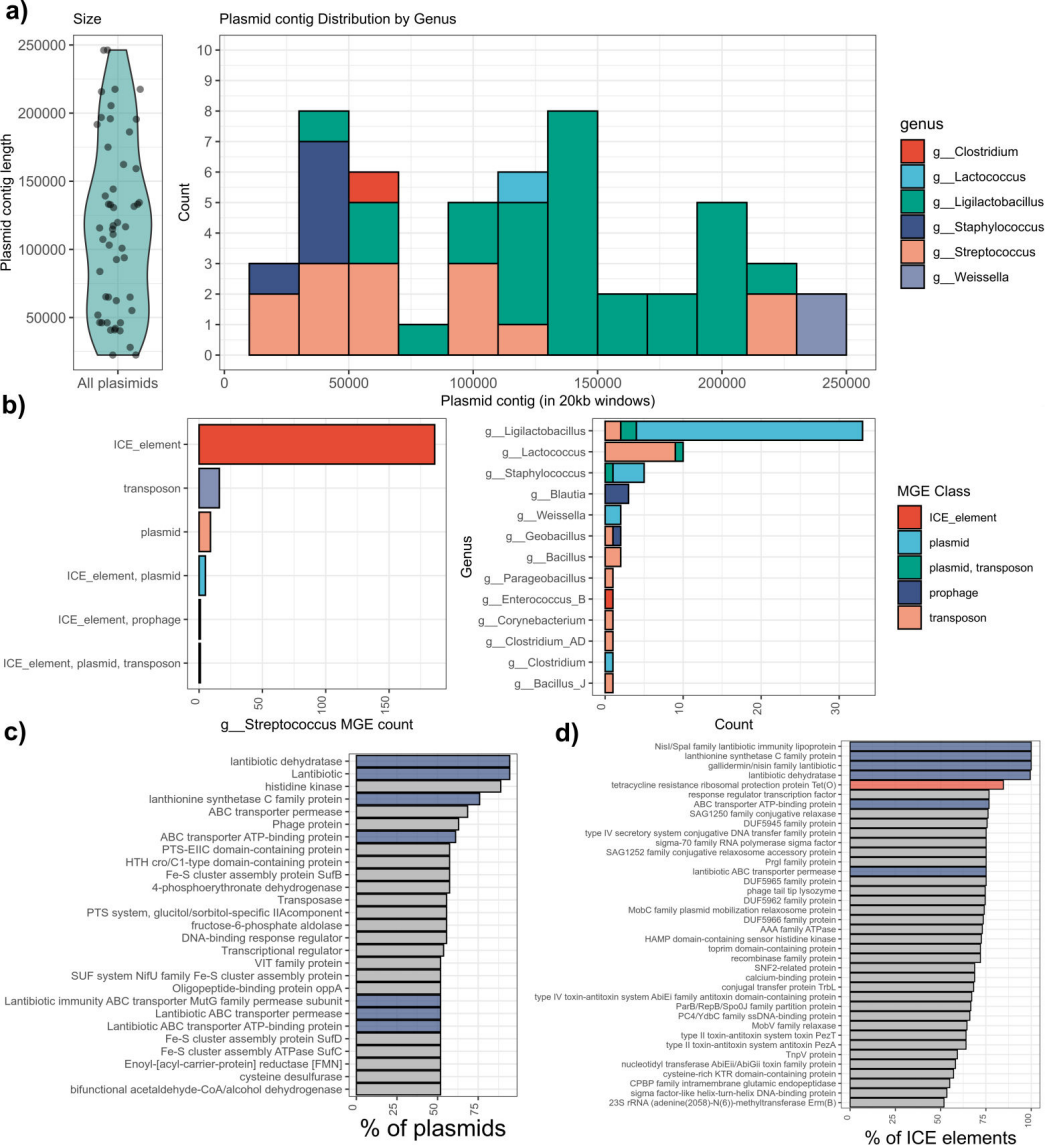
Nisin biosynthetic gene clusters are on mobile genetic elements (plasmids, transposons, and mobile prophage), and evidence of HGT exists. (**a**) A total of 287 nisin core peptides were identified on MGEs. Of these, 50 were predicted to be on plasmids. Shown on the left is the distribution of plasmid sizes. Shown on the right is the genus level order for each nBGC that is encoded on a plasmid. (**b**) The left plot is the total counts of MGE stratified by genus. The majority of ICEs carrying a nBGC are within the genus *Streptococcus*. The right plot shows all counts below 50 of the same data for clarity. In this plot, an “ICE_element, plasmid” is an ICE localized to a plasmid. “prophage, ICE_element” is an ICE localized within a predicted prophage. (**c**) The gene content of predicted plasmids as a percentage of plasmids containing gene products. (**d**) The gene content of predicted ICE as a percentage of ICE containing gene products.

As we observed the presence of multiple nBGCs shared between genomes, we wanted to investigate the presence of nBGCs on MGEs. A total of 281 nBGCs were identified on MGEs: 187 of these were predicted to lie within an ICE, 54 on plasmids, 34 on transposons, and 5 within prophage (Fig. 4a and b; Table S6). The nBGC encoded by *Enterococcus hirae* was predicted to be on a 61,771 bp T4SS-type ICE. The majority of MGEs were predicted within the genus *Streptococcus* with *S. suis* found to have the most nBGCs on ICEs (*n* = 138) (Fig. 4b). Invasive *S. suis* have been identified in Spain enriched with ICE (44). Similarly, there are multiple *S. suis* enriched in ICE encoding the BGC suicin (9). In total, 54 putative nBGCs were predicted to be located on plasmids. This included the plasmid pJOS1 (NZ_WHVU01000008.1), a nisin J-producing plasmid identified in *S. capitis* and plasmids in *Ligilactobacillus salivarius* (GCF_009865885.1), *Streptococcus pasteurianus* (GCF_033023195.1), *Streptococcus agalactiae* (GCF_000310565.1), and *Streptococcus ruminantium* (GCF_009731175.1). These plasmids were found to be more diverse in gene content compared to the predicted ICE (Fig. 4c). As previously mentioned, an nBGC was identified on a prophage genome. A total of five other nBGCs were identified on prophage. Three of these are in *Blautia* sp. and one is in *S. agalactiae*. It has been highlighted previously that multiple *S. suis* isolates harbor an ICE approximately 70 kb in length, harboring a suicin-like bacteriocin and the tetracycline resistance determinant *tet*(O) (9). This was observed in predicted nBGCs encoded on ICE, which were found to co-localize with toxin-antitoxin systems and the tetracycline resistance determinant *tet(O*), driven by these suicin-type nBGCs (Fig. 4d). A total of 142 out of 189 ICEs contain a type IV secretory system conjugative DNA transfer family protein, which plays a role in ICE dissemination. There are 44 ICEs that encode the bacitracin transport protein BcrA and 14 ICEs containing genes encoding the Tet40 tetracycline transporter. A full table of genes localized to ICE and plasmids with nBGCs is found in Tables S8 and S9, respectively. Of note are genes encoding tetracycline resistance ribosomal protection protein Tet(O) (*n* = 160), Erm(B) (*n* = 98), and APH(3')-IIIa (*n* = 13), which are responsible for resistance to tetracyclines, macrolides, and aminoglycosides, respectively. Tetracycline and bacitracin-zinc are two of the most widely used antibiotics in the pig industry (45). A recent study demonstrated that *sstFEG*, a NisFEG-like resistance determinant from *S. suis*, was also able to confer bacitracin tolerance (46). Therefore, we wanted to determine if NisI and NisFEG immunity systems from *L. lactis* could give tolerance to bacitracin. However, they were unable to confer bacitracin tolerance when expressed in *L. lactis* MG1614 (0-fold change in MIC value) (Table S10).

### Expression of novel nisin variants using *L. lactis* NZ9800

**Fig 5:**
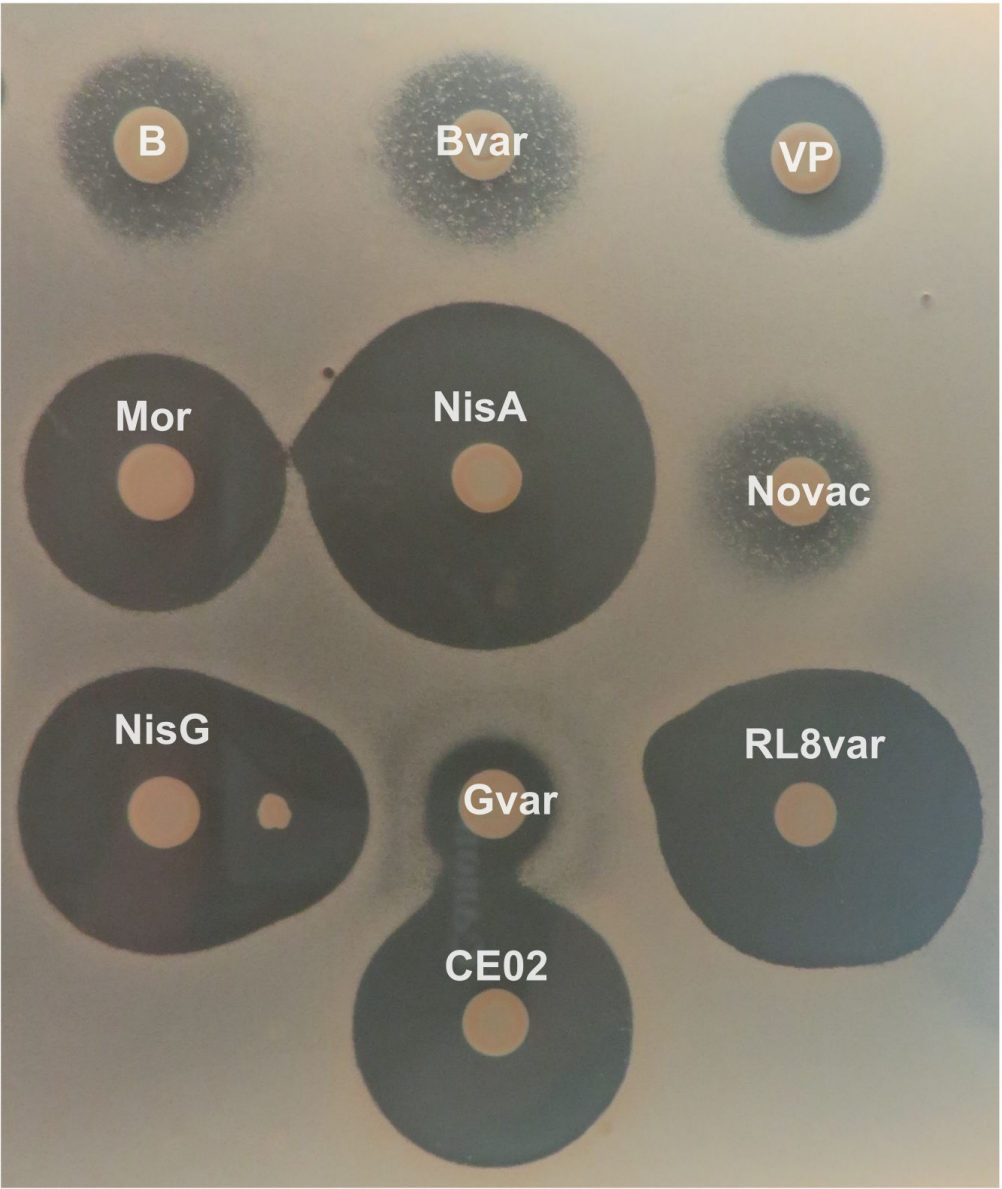
Heterologous expression of nisin peptides in *L. lactis* NZ9800. The following nisin variant peptides were heterologously expressed in *L. lactis* NZ9800: blauticin (B), a blauticin variant (Bvar), nisin VP, Moraviensicin (Mor), nisin Novac, nisin G (NisG), a variant of nisin G (Gvar), nisin RL8var, and CE02. Activity was determined with a thin overlay of MRS medium seeded with *L. bulgaricus* LMG6901. The expression nisin A (NisA) was used as a control.

We wanted to test whether predicted novel nisin variants were active and were capable of being heterologously expressed using the LanBC system from *L. lactis* NZ9800. A total of nine core peptide sequences were chosen based on the novelty of their sequences or the detection of the genes in commensal gut bacteria. Nisin Novac was chosen as it is a core peptide from the plant-associated bacterium *Nocardia vaccinii*, and only a single nisin variant has been described thus far from the phylum Actinomycetota (Flavucin, *Corynebacterium lipophiloflavum* DSM 44291) (47). However, no natural production has been observed within the phylum to date. Nisin VP was chosen as it is from a novel species, *Velocimicrobium porci*. A novel variant (nisin Gvar) was chosen given its high homology to the *Lb. salivarius*-derived nisin G that exhibits antimicrobial activity against *Fusobacterium nucleatum* (48, 49). A blauticin variant (nisin Bvar) was chosen as it was from the human gut anaerobe *Anaerobutyricum hallii*, a species lacking any evidence of antimicrobial activity. Nisin CE02 is potentially produced by *Clostridium* sp. E02 which was isolated from soil sediment in the River Dee, Liverpool (16). Nisin RL8var and nisin Gvar variants are located in *Lacrimispora* sp. *and Erysipelatoclostridium ramosum*, respectively. Blauticin (B), Moraviensicin (Mor), and nisin G (G) were also heterologously expressed to determine if these variants could be produced using the nisin A machinery. All nine peptides were expressed in *L. lactis* NZ9800, and all peptides exhibited antimicrobial activity (Fig. 5). Activity was determined as a zone of clearing when overlaid with the indicator strain *Lactobacillus bulgaricus* LMG6901 in a deferred antagonism assay. Masses of appropriate sizes are detected for all the heterologously expressed variants as seen in Table 1. *L. lactis* NZ9800 did not produce a zone against *L. bulgaricus* LMG6901 (data not shown).

**TABLE 1:** Colony MALDI TOF mass spectrometry analysis of *L. lactis* NZ9800 harboring the nisin variant genes on plasmid pCI372^*a*^

Peptide	−9 _H_20	−8 _H_20	−7 _H_20	−6 _H_20
Nisin A	–	**3,354**	3,372	3,390
Blauticin	3,154*	3,172	3,190	3,208
Bvar	3,126*	3,144	3,162	3,180
Nisin G	–	**3,404**	3,422	3,440
Nisin Gvar	–	3,422	3,440	3,458
Nisin Novac	**2,936**	2,954	2,972	2,990*
Nisin VP	**3,100**	3,118	3,136	3,152
CE02	–	**3,312**	3,320	3,338
RL8var	**3,162**	3,180	3,198	3,216*
Moraviensicin	–	**3,344**	3,362	3,380

^*a*^ Bold font indicates the expected mass of the fully modified peptide. MALDI-TOF reveals the extent of modification of each variant given that the essential dehydration of threonines and serines by NisB can easily be detected (18 Da reduction per dehydration). *Not detected. "–" denotes not applicable.

### Nisin VP is a novel natural nisin variant from *Velocimicrobium porci* WCA-693-APC-MOT-I

**Fig 6:**
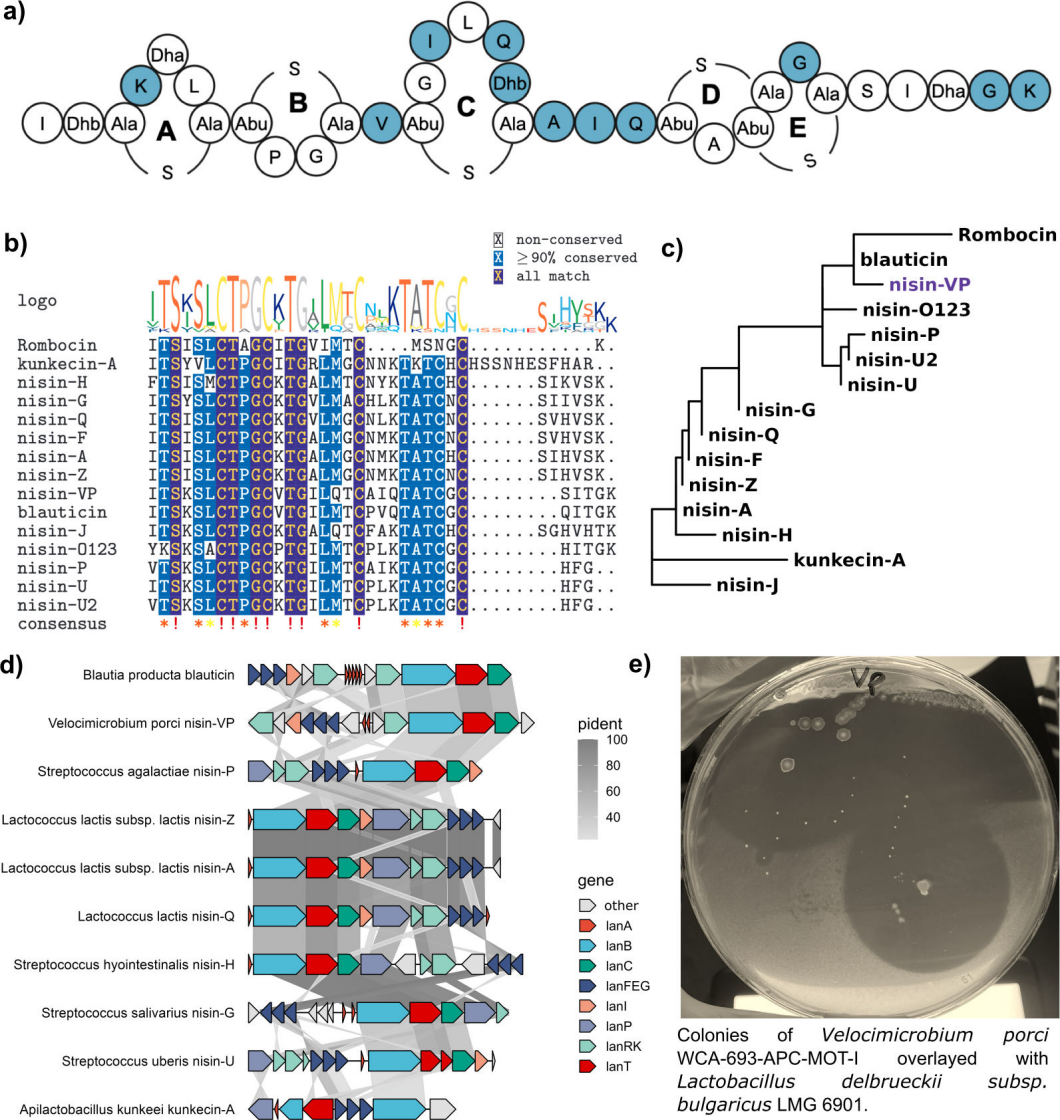
Nisin VP is a novel natural nisin variant produced by *Velocimicrobium porci* DSM 107250. (**a**) The putative structure of nisin VP. The peptide has all five nisin rings and has four dehydrated residues. Each residue in blue highlights differences from nisin A. The core has a novel “AIQ” hinge region. Post-translational modifications are indicated as follows: Abu: 2-aminobutyric acid, Ala-S-Ala: lanthionine, Abu-S-Ala: 3-methyllanthionine, Dha: dehydroalanine, Dhb: dehydrobutyrine. (**b**) A multiple sequence alignment (MSA) of nisin VP with known nisin variants. (**c**) A phylogenetic tree of the same sequences in the MSA. The construction of the tree was subjected to 200 bootstrap replicates. Nisin VP is most similar to blauticin, which is another gut-derived nisin. (**d**) A synteny plot of nBGCs that encode characterized natural nisin variants. Of note is the absence of a LanP in the operon for nisin VP. Only shared amino acid identity greater than 20% is shown with an *E*-value of 1e-05. (**e**) Antimicrobial activity of *Velocimicrobium porci* against *L. bulgaricus* LMG6901.

*Velocimicrobium porci* WCA-693-APC-MOT-I was obtained from the Leibniz Institute DSMZ German Collection of Microorganisms and Cell Cultures GmbH. It is a Gram-positive, strictly anaerobic, motile, low G + C content (34.9%), and fast-growing bacterium (50). The bacterium was isolated from the faeces of a 20-week-old pig and is a member of the family *Lachnospiraceae* (50). The bacterium was chosen as it is a representative of a new genus that encodes a 3,100 Da peptide consisting of 33 amino acids, which we term “nisin VP,” and displays antimicrobial activity as determined by heterologous expression (Fig. S4). The peptide has residues that would facilitate the formation of the characteristic nisin five-ring structure (including four dehydrated residues at positions 2, 5, 18, and 31), but with 11 amino acid changes compared to nisin A (Fig. 6a). The peptide was found to have a completely novel “hinge” region AIQ. The core peptide is most similar to nisin O and blauticin (Fig. 6b and c), both of which are gut-derived nisin variants. Of note is the absence of a protease within the nBGC (Fig. 6d). We identified three putative NisP-type serine proteases in the *V. porci* genome (WP_154515492.1, WP_154516573.1, and WP_154518886.1) (51). Two of which are predicted to be extracellular proteases as seen with subtilin produced by *Bacillus subtilis* (51). Initially, the bacterium did not show antimicrobial activity, however, after culturing the bacterium on plates seeded with Nisaplin (0.01 µg/mL nisin, GAM^nisin+^), followed by subculturing onto a new plate (GAM^nisin-^), we observed production (Fig. 6e). This assay was then repeated to check for activity in the cell-free supernatant, and activity was only observed from the induced culture (Fig. S5a). Colony mass spectrometry confirmed a 3,100.27 Da mass (Fig. S5b). The genome did not contain other BGCs that could produce a peptide with the same mass as predicted by AntiSMASH7 and BAGEL4 (52, 53). As we observed that nisin A (from Nisaplin) could induce production of nisin VP, we wanted to test whether nisin VP could equally induce the nisin A two-component regulatory system NisRK from *L. lactis*. However, only nisin A, and not nisin VP, was able to induce GFP (Nisin promoter) expression in *L. lactis* NZ9000 between 0.5 and 20 ng/mL (Fig. S6).

## DISCUSSION

In this study, we identified genes encoding 1,060 nisin putative prepropeptides across 915 genomes. This suggests that the ability to produce nisin-like peptides is more widespread than previously acknowledged. We discovered genes encoding nisin-like peptides in 5 phyla, 25 families, 59 genera, and 123 species. Of note is the presence of nBGCs in bacteria that are designated as human pathogens, such as *S. suis*, *S. agalactiae* ST27 and ST19, and *S. aureus* (Fig. S2; Table S5) (54). We also heterologously expressed a nisin variant from the phylum Actinomycetota. This phylum has been predicted to be a source of novel lanthipeptides but has been underexplored with respect to bacteriocin production to date (36, 55). *Nocardia vaccinii* NBRC 15922 was previously predicted to be the only member outside the Bacillota to encode a nBGC. This study discovered 35 genomes within the phylum Actinomycetota encoding a nisin-like BGC vastly expanding the biosynthetic potential of the phylum (36). The class I lanthipeptide microbisporicin (NAI-107) produced by *Microbispora* sp. has two active peptides between 2,246 and 2,230 Da and is one of the most potent lanthipeptides (56, 57). Using rapidly expanding databases, we have identified nBGCs in species without previously described nisin production, such as *Bifidobacterium infantis* JCM 11347, *Tsukamurella paurometabola* DSM 43341, and *Corynebacterium freiburgense* DSM 45254. nBGCs were also observed in the phyla Chlamydiota and Bacteroidota. *Tsukamurella paurometabola* (DSM 43341) is a clinical isolate that encodes an nBGC with a core peptide with 58% similarity to nisin A. Members of the genus *Tsukamurella* are considered to be emerging pathogens with an underestimated prevalence due to clinical features resembling *Mycobacterium tuberculosis* (58).

Yet another observation is the low degree of nBGC synteny between taxa, such as those encoding nisin VP and nisin A. The nBGC from *V. porci* lacks a gene encoding a protease. It is notable that a fully active peptide is produced by the strain. Similarly, the operon is split in terms of open reading frames. We found that core peptides, as expected, are highly conserved with respect to amino acids involved in ring structures but do contain highly variable regions. The C-terminus of the peptide is the most variable. It has been shown that this region of the peptide is crucial for determining the efficiency of the immunity-associated LanFEG efflux systems, as truncated peptides lacking this region up to the last residue of ring E exhibit a 3.4-fold reduction in protection (59). Positions 4, 12, and 15 are the most variable residues within rings A, B, and C. Six unique peptides were found to have an alternative lipid II binding site, “CTAGC,” which resembles that of rombocin (10). These include an nBGC encoded in *Streptococcus thoraltensis* (WP_247213748.1, GCA_932751155.1) and multiple nBGCs encoded among *S. suis* isolates. All nine peptides showed antimicrobial activity when heterologously expressed, though activity varied likely due to bias in our *L. bulgaricus* LMG6901 screen.

Although LanB and LanC proteins differed in amino acid identity, they shared highly conserved predicted protein structures (Fig. 2d). Alanine scanning of the NisB enzyme performed by others highlighted several key amino acids for its activity. These include arginine residues at positions 14, 83, 299, and 464 and threonine 89 in the N-terminal end of the protein (60–62). These residues are all conserved in the nisin VP LanB enzyme despite their low overall amino acid identity (Fig. S3). The diversification of gene and protein sequence identity could be due to codon usage bias, overall GC content, and tRNA preference leading to species-specific “domestication” of nBGCs (63). Nisin Novac and nisin VP LanBs share very low amino acid identity to NisB, but their cognate peptides were still modified by the NisB, NisC, and NisT machinery in *L. lactis* NZ9800. Although modified active peptides were produced*,* we observed peptide masses that indicated inefficient dehydration of residues (Table 1). This phenomenon has also been observed for heterologous expression of other nisin variants in *E. coli* but also for nisin A in *L. lactis* (16, 64).

*Velocimicrobium porci* is a single isolate representing the genus *Velocimicrobium*. The bacterium was isolated from faeces as part of a study focused on the functional and taxonomic diversity of the pig gut microbiome (50). The isolate shares less than 50% shared genes with its closest phylogenetic neighbor, *Acetivibrio ethanolgignens* (50). Nisin VP was only produced by *V. porci* when initially grown on a low concentration of nisin A. This suggests that the peptide can be induced by nisin A through its two-component signal transduction system (RK). However, we did not observe induction of the nisin A promoter using nisin VP. Another interesting observation is the presence of two Stage 0 sporulation A-like proteins within the nisin VP operon and a putative autoinducer peptide, which suggests the regulation of the operon might be linked to quorum sensing and sporulation as seen in *Bacillus* sp. and sporulation (65). The operon was not predicted to be on an MGE, but it is located in a region of approximately 50 kb flanked by tRNA genes and encoding an excisionase and phage protein. This suggests that the operon may have been gained through a phage-mediated HGT event.

If nisin is to be used as an alternative to antibiotics, it is vital that we understand the distribution of gene-encoded resistance determinants and nBGCs. There is increasing evidence of resistance and tolerance gene determinants, such as the bacitracin efflux system *bceAB* and *lanFEG* conferring nisin resistance (16, 19, 46, 66–68). We found that 27.5% of sources were related to pigs. Notably, the nisin H producer, *S. hyointestinalis* DPC6484, was also isolated from a porcine source. A recent study highlighted the presence of suicin, an nBGC encoded by *S. suis* 90-1330 on an excisable ICE that co-localizes with genes for tetracycline resistance and erythromycin resistance (9). A similar ICE, ICESsuCZ130302, was also shown to confer multidrug resistance, where transconjugants showed increased resistance to tetracycline, neomycin, and erythromycin (69). This highlights the necessity to catalog bacteriocin production genes on MGEs and their genetic cargo. Tetracycline has been widely used in pigs for growth promotion and the treatment of gastrointestinal infections (45). In 96 swine and human *S. suis* isolates from China, all were tetracycline-resistant, with resistance localized to an ICE in 10 cases and transferable at a frequency between 1.31e-08 and 5.14e-08 (70). However, the gene content of the ICE was not discussed. An investigation into invasive *S. suis* isolates in Spain found that ICEs contributed to the AMR gene content of isolates, one of which had a *bceAB* resistance determinant co-localized with tetracycline resistance (44).

In this study, we observed 44 ICE elements harboring both an nBGC and bacitracin resistance. Given the link between nisin production on *S. suis* ICEs and FEG-like resistance, we tested if NisFEG conferred bacitracin tolerance but found no change in MIC (Table S10). However, a limitation of this test was that bacitracin was used alone, whereas a separate study found bacitracin supplemented with 1 mM ZnCl_2_ to be more potent due to increased access to the membrane (71). Other bacitracin resistance mechanisms have been identified in *Streptococcus mutans* UA159, *Clostridioides difficile*, and *Listeria monocytogenes*, and these can confer nisin resistance (67, 72, 73). While bacitracin resistance is linked to nisin resistance, evidence that nisin immunity or resistance genes protect against bacitracin is limited (46). Spontaneous bacitracin-resistant mutants of *L. lactis* can produce more nisin, providing further evidence of a link between the two antimicrobials (17). A novel ABC transport system *sstFEG* has been shown to increase bacitracin tolerance in *S. suis* and is encoded beside *nisI* and *bceAB* downstream from a nBGC (46). Bacitracin is a cyclic peptide antibiotic that has been banned as a commercial feed additive in Europe since 1999 (74). However, it has only been withdrawn from use as a growth promoter in China since 2020 (75). It is plausible to suggest that the use of bacitracin as a porcine feed additive may have driven selective pressures towards the acquisition of nisin immunity and resistance mechanisms—a tale that mirrors the use of avoparcin and the onset of vancomycin resistance among enterococci (76).

This study has shown that nisin production machinery and immunity systems are more widespread than previously appreciated. Some of these potential gene clusters are found in pathogenic bacteria that have sequence types of etiological concern and are localized on MGEs. We demonstrate that *V. porci* is a novel natural producer of nisin VP. This diversity of production has important implications for the use of nisin in altering microbial ecosystems, where its application can select for resistant subpopulations or apply selective pressure for HGT. There is increasing evidence pointing towards a therapeutic role for nisin in tackling AMR. Therefore, discovering the frequency and variation of the genes responsible for its production and immunity may be a critical factor in deciding strategies to apply nisin as a biotherapeutic.

## MATERIALS AND METHODS

### Genome mining

The nr database and MGnify gut databases were downloaded in FASTA format (15-Nov-23). Nisin-like core peptides were extracted using the regular expression “..S.S.CT..C.[TS].{1,6}C.{1,4}[TS].{1,2}[TS]C.{1,3}C,” where “.” denotes any amino acid. A total of 58 nisin-like core peptides were identified, and these were used to train a HMM model which was then searched back into the nr database and MGnify databases with an *E*-value cutoff of 0.005 with a subset of sequences manually looked at over different *E*-values to select this threshold. The MAGs downloaded from MGnify were found to have below 4.8% contamination and above 59.6% completeness. The InterPro family for LanB N-terminal dehydratase was downloaded and aligned using MUSCLE v5.01 with the option “super-5” (77). HMMER v3.3.1 (78) was used to search the nr database for LanB-type proteins using hmmsearch. An *E*-value of 1e-05 was chosen to balance specificity and sensitivity (79). Rodeo2 was used to retrieve the genomic context of candidate LanB-type proteins with the options “-j 8 min 30 -max 100 -o 25 ft 'cds' -fn 20 -ex -ea” (80, 81). The nisin-like HMM model was also used to search the rodeo output for nisin-like prepropeptides.

### Phylogenetic tree of producing organisms

Genome assemblies found in GenBank containing putative nBGCs were downloaded using NCBI data sets v16.10.1 (82). Phylogenetic trees were constructed using GTDB-Tk v2.4.0 with the parameters “classify_wf” and “--skip_ani_screen” (38). The final tree was constructed using FastTree v2.1.11 within the GTDB-Tk (83). Metadata was downloaded using data sets and filtered to include “geo_loc_name,” “isolation_source,” “host,” and “collection date,” and was manually curated. Hmm, models were created for the nisin machinery LanB, LanC, and LanI. These were searched back into the protein content of the genomes to determine the presence and absence of core machinery. This was then overlayed on the plotted tree using R package ggtree v3.18 (84).

### Detection of plasmids, prophage, and AMR genes

Plasmids were predicted using Platon v1.6 with the “accuracy” settings (85). Platon was chosen for its ability to predict plasmids in isolate genomes to a high degree of accuracy. The tool also looks for key plasmid-specific proteins using sensitive HMM models and checks for circular contigs. Prophage was predicted using PhiSpy v4.2.21 and PHASTEST v3.0, and completeness of prophages was determined using CheckV v1.0.1 with the setting “end_to_end” (86–88). Only “high quality” prophage with >90% completeness was brought forward for analysis in the data set. ICEs were predicted using ICEfinder v2.0 with default settings and the parameter “-t Single” for single genome inputs (89). However, ICEfinder was unable to detect conjugative transposons in the genus *Lactococcus*. Therefore, in order to predict transposon-containing nBGCs missing from our data set, we used Tncomp_finder v1.0.0 to find insertion sequences (IS) (https://github.com/danillo-alvarenga/tncomp_finder). Regions within each genome that contained the same ISs flanking an nBGC, with total length between 10 and 130 kb, were considered putative composite transposons. These were then curated to include a transposase encoded within the 120 kb window. AMR genes were predicted using AMRFinderPlus v3.11.26 (90). In order to gain insight into the gene content of these MGEs, we standardized the annotations using Bakta v1.9 using database v5.0 (91). Bar charts were plotted using ggplot2 using R v4.3.0.

### Core peptide analysis

Core peptide sequences were made non-redundant using tool SeqKit v2.0.0 with the command “rmdup -s” (92). This data set was then manually curated to remove leader sequences using canonical leader sequences “GASPR” and “PQ.” Where a leader sequence could not be determined, the peptide was aligned to its closest relative with a leader sequence and predicted accordingly. The peptides were aligned using MUSCLE v5.1 (77). A sequence similarity network (SSN) of the core peptides only was constructed using the Enzyme Function Initiative-Enzyme Similarity Tool (EFI-EST) with an *E*-value cutoff of 1e-15, and the corresponding network was visualized in Cytoscape v3.10.1 with the “prefuse force directed layout” (93). Known characterized nisin variants were manually colored. To determine the most highly conserved residues across all peptides found, we used MEME Suite v5.4.1 (94). It was run using the settings “-p 1 -allw -minw 10 -protein -nmotifs 1 -alph meme.alph,” where “meme.alph” is a custom color palette. For comparing Nisin VP to known nisin variants, core peptides were manually truncated to remove the leader sequence. They were aligned using muscle v5.1 (77) and visualized using the R package msa v1.22. Raxml-ng v1.2.0 was used to construct a phylogenetic tree with the options “--all --seed 2—model LG --bs-trees 200 --bs-metric tbe,fbp” (95). The phylogenetic tree was constructed using ggtree v3.18 (84). Genus-level taxonomy was appended to the Rodeo output table of nisin-like bacteriocin positive accessions using the R package “taxonomizer” (https://cran.r-project.org/web/packages/taxonomizr/). The same protein accession of a core peptide shared among different genera indicates that the prepropeptide is shared at 100% amino acid identity. The Sankey plot was created using a shared protein accession for a core peptide and genera as the input. Core peptides occurring more than 10 times were included and plotted in a Sankey plot using “ggsankey” (https://github.com/davidsjoberg/ggsankey).

### Operon synteny

BLASTp v2.11.0+ was used to perform all-vs-all proteins comparisons, and synteny with BLAST links was plotted using gggenomes (v0.9.12.9) (96).

### Percentage ID matrix

Proteins were identified as hits to the LanB and LanC HMMs and deduplicated using SeqKit v2.0.0 with the command “rmdup -s” (92). A percentage identity matrix was calculated using the pairwise global aligner from Biopython v1.83.

### Structure prediction and analysis

The structures for the nisin A machinery were downloaded on 22 February 2024 from the Protein Data Bank (PDB) (https://www.rcsb.org). Protein structures were imported into PyMOL and were colored as follows: “spectrum b, red_yellow_green_cyan_blue, minimum = 50, maximum = 90, set_color n0, [0.051, 0.341, 0.827] set_color n1, [0.416, 0.796, 0.945] set_color n2, [0.996, 0.851, 0.212] set_color n3, [0.992, 490, 0.302] color n0, b < 100; color n1, b < 90 color n2, b < 70; color n3, b < 50, spectrum b, rainbow_rev, minimum = 0, maximum = 100.” This color setting mirrors the AlphaFoldDB confidence score threshold. Protein structures were predicted using Alphafold2 v2.3.2 with the setting “monomer” (97). The highest-ranking structures were used for comparisons and visualized in Pymol v2.5.8. Predicted local distance difference test (pLDDT) of each structure was plotted using R v4.3.0, and RMSD was calculated using the R package bio3d v2.4.1. TM-align v20170708 was also used to compare protein 3D structure (98).

### Cloning

Core peptide genes were synthesized (Genewiz Azenta Life Sciences, Leipzig, Germany) and inserted into a pUC plasmid. The genes were subsequently cloned into the vector pCI372 as described previously by Field et al. (99) using the primers outlined in Table S12 of the supplementary data and transformed into *E. coli* Top 10 (Invitrogen) cells (99). Plasmids from positive transformants were sequenced (Genewiz Azenta Life Sciences, Leipzig, Germany) using the primers pCI372For and pCI372Rev to verify integrity of the construct. Verified plasmids were transformed into *L. lactis* NZ9800 and grown on GM17 agar containing 10 ug/mL chloramphenicol (Oxoid, Waltham, MA, USA). Positive clones were cultured on nisin M-containing media to induce nisin variant production. This nisin variant holds induction capacity with no antimicrobial activity (100).

### Mass spectrometry

Matrix-assisted laser desorption/ionization Time-of-Flight (MALDI-TOF) mass spectrometry was performed on *Velocimicrobium porci* WCA-693-APC-MOT-I. Briefly, colonies were mixed with 50 µL propan-2-ol 0.1% TFA, vortexed three times, and centrifuged at 16,000 × *g* for 30 s. MALDI TOF mass spectrometry was performed using an iDPlus Performance MALDI TOF mass spectrometer (Shimadzu Europa GmbH, Duisburg, Germany). An aliquot (0.5 µL) of matrix solution (α-cyano 4-hydroxy cinnamic acid, 10 mg mL^−1^ in acetonitrile–0.1% [vol/vol] trifluoroacetic acid) was deposited onto the target and left for 20 s before being removed. The residual solution was allowed to air-dry, 0.5 µL of the sample solution was deposited onto the pre-coated sample spot, and 0.5 µL of matrix solution was added to the deposited sample and allowed to air-dry. Samples were then analyzed in positive-ion linear mode.

### Protein purification

The nisin variant-producing strain *L. lactis* NZ9800 pCI372-*nis*VP was inoculated (1% fresh overnight culture) into 2 L of tryptone yeast (TY) broth (Merck, Kenilworth, NJ, USA), supplemented with glucose (0.5% [vol/vol]) (Sigma-Aldrich, USA), β-glycerophosphate (Sigma-Aldrich, USA) (2% [vol/vol]), and 20 ng/mL nisin (Nisaplin, Danisco, DuPont), and incubated for 16–18 h. The sample was then centrifuged at 8,000 × *g* for 20 min. The cell-free supernatant (CFS) was passed through 60 g of pre-equilibrated Amberlite XAD16 beads (Sigma-Aldrich, USA) and washed with 500 mL of 30% ethanol and eluted in 500 mL of 70% isopropanol (IPA) (Fisher Scientific, Waltham, MA, USA) with 0.1% trifluoroacetic acid (TFA) (Sigma-Aldrich, St. Louis, MO, USA). In tandem, the cell pellets were resuspended in 250 mL of 70% IPA–0.1% TFA, stirred for 3 h at room temperature, and then centrifuged as described above. The supernatants were combined and were concentrated through rotary evaporation (Buchi, Switzerland) to approximately 220 mL, adjusted to pH 4.0, and passed through a Phenomenex SPE C-18 column to a final volume of 80 mL. Twelve milliliters was further concentrated by rotary evaporation to 2 mL and purified through HPLC using a Phenomenex C12 Reverse-Phase (RP) HPLC column (Jupiter 4 µ proteo 90 Å, 250 × 10.0 mm, 4 µm) in a gradient of 25%–60% acetonitrile (Fisher, Waltham, MA, USA) containing 0.1% TFA. The relevant fractions were collected, pooled, and freeze-dried (LABCONCO, Kansas City, MO, USA).

### Agarose and agar well diffusion assay (WDA)

Deferred antagonism agar-based assays were performed to assess the bioactivity of the nisin derivatives blauticin, Bvar, CE02, nisin novac, nisin G, nisin Gvar, Moraviensicin, RL8var, and nisin VP as expressed by *L. lactis* NZ9800. Briefly, fresh overnight cultures of each *L. lactis* NZ9800 test strain were replicated on GM17 agar plates containing 50 ng/mL nisin M using a 96-pin replicator (Boekel, PA, USA) and incubated overnight at 30°C (100). Following ultraviolet radiation treatment for 50 min, the plates were then overlayed with GM17/mMRS agar (0.75% agar) seeded (0.5% inoculum) with either *L. lactis* HP or *L. bulgaricus* DSM 6901 indicator organisms. Plates were incubated overnight in appropriate conditions (37°C and anaerobically for *L. bulgaricus* DSM 6901, and 30°C aerobically for *L. lactis* HP strains) and then examined for the presence of inhibitory zones.

### Antimicrobial activity of *Velocimicrobium porci* and BGC annotation

*Velocimicrobium porci* WCA-693-APC-MOT-I was obtained from DSMZ and routinely grown on pre-reduced Gifu anaerobic medium (GAM) (https://ie.vwr.com/store/product/40101266/gifu-anaerobic-broth) under strict anaerobic conditions maintained by a Type A vinyl Coy Lab anaerobic chamber. The strain was plated on 0.01 µg/mL final concentration nisin. Nisin was added in the form of Nisaplin, which is 2.5% nisin. The bacterium was then sub-cultured onto a GAM plate without Nisaplin and overlayed with *L. bulgaricus* LMG6901 to determine antimicrobial activity. Activity was only observed after subculturing from a plate seeded with Nisaplin. The whole-genome sequence of *Velocimicrobium porci* WCA-693-APC-MOT-I (GCF_009696045.1) was downloaded from NCBI. AntiSMASH v7.1 and BAGEL4 were used to predict putative BGCs (52, 53). Where a BGC was predicted, the core peptide was manually inspected to determine potential cleavage sites and modifications, where relevant. However, no predicted masses were observed that would pertain to a protein/peptide 3,100 Da in weight. To identify putative LanP proteases, we searched the protein content of *V. porci* using the conserved D-motif (HGTHVAG), H-motif ([IV]D[ST]G), and S-motif (G[TN]S.A) found in this class of peptidases, where amino acids within a square bracket represent alternatives and a dot denotes any amino acid. This resulted in three potential serine proteases identified in the *V. porci* genome that are involved in the maturation of the core peptide (WP_154515492.1, WP_154516573.1, and WP_154518886.1). PSORTb v.3.0 predicted that two of these peptidases were localized extracellularly (WP_154516573.1 and WP_154518886.1, score = 9.7) with the third unknown (WP_154515492.1, score = 2.5) (101).

### Assessment of purified nisin A and nisin VP induction capacity using a green fluorescent protein (GFP) reporter system

The induction capacity of nisin VP and native nisin A was assessed using a green fluorescent protein (GFP) assay. Cells from a fresh overnight of *L. lactis* NZ9000pNZ8150*gfp*+ were diluted 1:100 in fresh GM17 and incubated until the absorbance (A600) reached 0.5, then further diluted to reach a final concentration of 10^5^ CFU/mL. Nisin VP or nisin A was added to reach a final concentration range of between 0.5 and 20 ng/mL in a black 96-well plate containing 100 uL of the culture. Green protein fluorescence was detected in terms of relative fluorescence units (RFU) with a Synergy HTX (BioTek, Agilent, USA) with excitation and emissions filters set to 485 and 538 nm. GFP reporter data in relative fluorescence units (RFU) was plotted using GraphPad Prism v8.0.

### Minimum inhibitory concentration (MIC)

MIC assays were performed in triplicate in 96-well microtiter plates (Sarstedt, Newton, NC, USA) as previously described (18). Test indicator strains (*L. lactis* MG1614, *L. lactis* MG1614 pNZ-*nis*I, *L. lactis* MG1614 pNZ-*nis*FEG, and *L. lactis* MG1614 pNZ-*nis*IFEG) were grown overnight in appropriate media and conditions and were subsequently sub-cultured into fresh media and grown to reach an optical density of ~0.5 (OD600). The cultures were then diluted to a final concentration of 10^5^ CFU mL^−1^ in a volume of 1 mL. The antibiotic bacitracin (Sigma-Aldrich, St. Louis, MO, USA) was adjusted to a starting concentration of 100 µg/mL, and an aliquot of 100 µL was added to the first well, mixed, and then, 2-fold serial dilutions were made in GM17 broth. Subsequently, 100 µL of target strain was added to each test well, and the plates were incubated at 30°C for 16 h. The MIC was read as the lowest peptide or antibiotic concentration causing inhibition of visible growth.

## Data Availability

Scripts and methods used in this study are stored in https://github.com/DEHourigan/nisin_paper_2024.

## SUPPLEMENTAL MATERIAL

Supplemental material mbio.01545-25-s0001.pdf Captions for Tables S1 to S9; Fig. S1 to S6; Tables S10 to S12.

Table S1 mbio.01545-25-s0002.csv Genomes, isolation source, host, and taxonomy.

Table S2 mbio.01545-25-s0003.csv Core peptides and their genomic location.

Table S3 mbio.01545-25-s0004.csv Prepropeptides only identified by Rodeo.

Table S4 mbio.01545-25-s0005.csv Known nisin variants with characterized activity seen in the literature.

Table S5 mbio.01545-25-s0006.csv ST of all assemblies.

Table S6 mbio.01545-25-s0007.csv Mobile genetic elements.

Table S7 mbio.01545-25-s0008.txt Nisin-like core peptides.

Table S8 mbio.01545-25-s0009.csv Products encoded on plasmids.

Table S9 mbio.01545-25-s0010.csv Products encoded on ICE.

ASM does not own the copyrights to Supplemental Material that may be linked to, or accessed through, an article. The authors have granted ASM a non-exclusive, world-wide license to publish the Supplemental Material files. Please contact the corresponding author directly for reuse.
