# Supplementary material for: Nisin-like biosynthetic gene clusters are widely distributed across microbiomes
Source: mBio. 2025 Sep 10;16(10):e01545-25. doi: 10.1128/mbio.01545-25 (PMC12506125; doi:10.1128/mbio.01545-25)
Supplement: Supplemental material — Captions for Tables S1 to S9; Fig. S1 to S6; Tables S10 to S12. [file mbio.01545-25-s0001.pdf]

Nisin-like biosynthetic gene clusters are widely distributed across microbiomes – Supplementary Text

David Hourigan[1,2], Des Field[1,2], Ellen Murray[1,2], Ivan Sugrue[1,2], Paula M. O'Connor[1,3], Colin Hill[1,2], R. Paul Ross[1,2,3]

[a] APC Microbiome Ireland, Biosciences Institute, Biosciences Research Institute, College Rd, University College, Cork, Ireland

[b] School of Microbiology, University College Cork, College Rd, University College, Cork, Ireland

[c] Teagasc Food Research Centre, Moorepark, Moorepark West, Fermoy, Co. Cork, Ireland

Running Head: Nisin-like genes are widespread

#Address Correspondence: p.ross@ucc.ie

SUPPLEMENTARY FILE LEGEND

Supplementary Table S1 – This table contains the information for genomes in this study. The table contains the following columns: assembly, domain, phylum, class, order, family, genus, species, isolation_source, host_attribute_value, country_value. The columns isolation_source, host_attribute_value, country_value were manually curated from NCBI metadata.

Supplementary Table S2 – This table contains the sequences for the putative nisin-like core peptides found in this study. The table contains the following columns: nucleotide_acc, locus_tag, start, stop, sequence, id, assembly, domain, phylum, class, order, family, genus, species.

Supplementary Table S3 – This table contains the core peptides which were identified by Rodeo only. The table contains the following columns: Query, Genus/Species, Nucleotide_acc, start, end, dir, AA_seq, id.

Supplementary Table S4 – This table contains sequences for nisin-like core peptides with described antimicrobial activity. This includes both natural and synthetic constructs. The table contains the following columns: sequence, nisin_name

Supplementary Table S5 – This table contains sequence types (ST) for genomes used in this study. Column 1 contains the genome accession, Column 2 contains the ST species group, column 3 contains the ST and column 4 to 13 contain genes contributing to ST classification. A “-” denotes a null value.

Supplementary Table S6 – This table describes the nisin-like BGCs which are encoded on mobile genetic elements (MGE). This table contains the following columns: nucleotide_acc, detail, mge_class, genus, species.

Supplementary Table S7 – This FASTA file contains the unique nisin-like core peptides.

Supplementary Table S8 – This table describes the protein content of the plasmids which encode a nisin-like BGC. The table contains the following columns: product, contig_count.

Supplementary Table S9 – This table describes the protein content of the integrative and conjugative elements (ICE) which encode a nisin-like BGC. The table contains the following columns: product, seq_id_count.

Supplementary Table S10 - MIC table for bacitracin and Nisin A for the Nisin A machinery NisI, NisFEG and NisIFEG.

Supplementary Table S11 - Table of amino acid sequences of nisin-like peptides and the source.

Supplementary Table S12 - Primers used for cloning of nisin-like peptides.

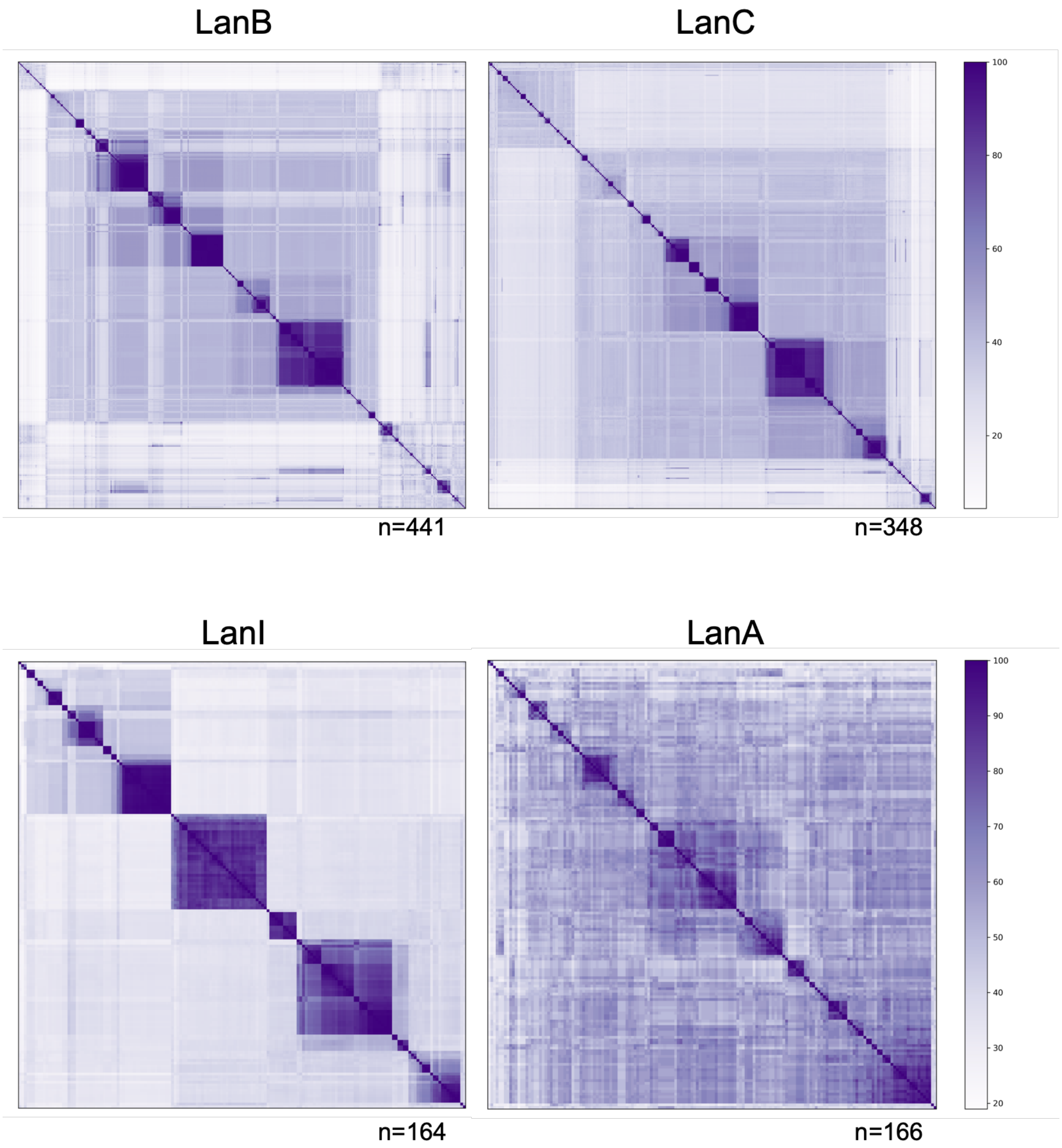

Fig. S1. A percentage identity matrix for LanB, LanC, LanI and LanA for all proteins found in this study.

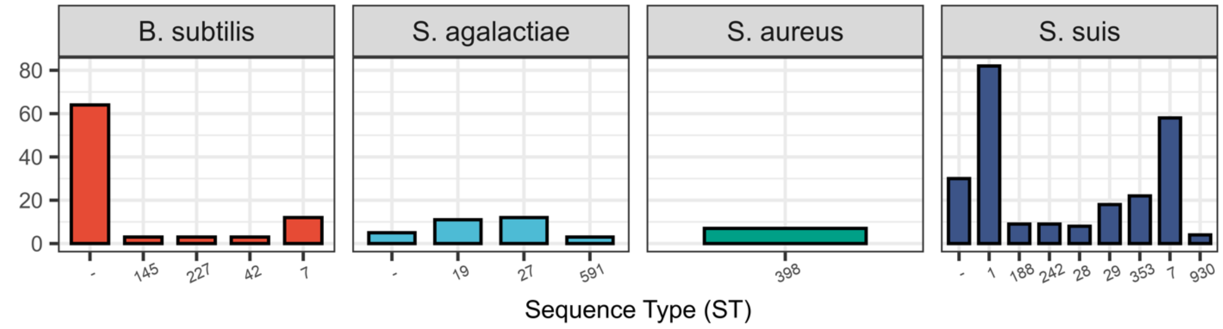

Fig S2. Sequence typing of strains. The plot shows a bar chart for genomes that had 2 or more sequence types present. *S. aureus* ST25 and ST398 are mainly Methicillin-sensitive Staphylococcus Aureus (MSSA), but can be MRSA between 5-7% but the latter ST398 is associated with increased biofilm formation. *S. agalactiae* ST19 is associated with oral biofilm formation. *S. suis* ST29 is an emerging pig pathogen and often encode IgM proteases that can aid persistence in piglets and high survival in porcine blood. *S suis* ST1 represents pathogenic MDR strains often encoding resistance to oxytetracycline, doxycycline, erythromycin, tylosin, and lincomycin (Rieckmann *et al.*, 2018; Wu *et al.*, 2023).

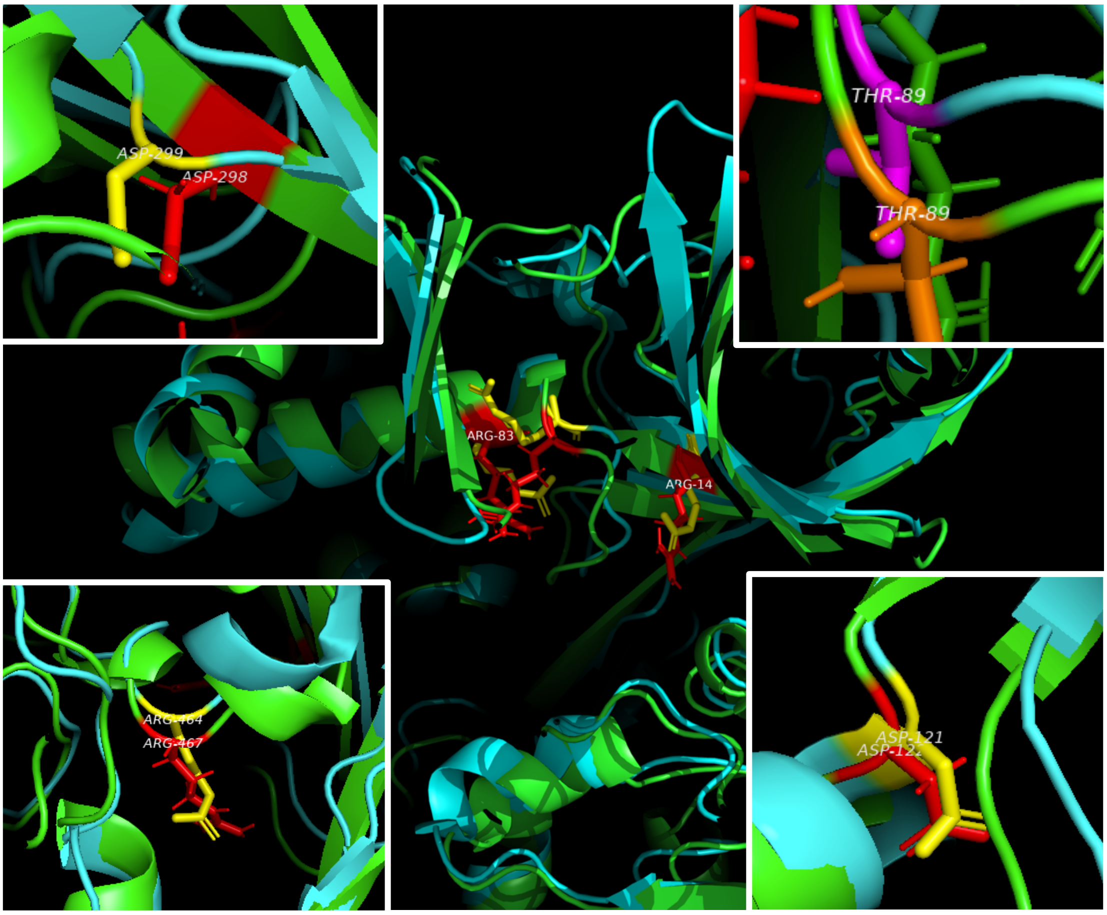

Fig. S3. N-terminal region of LanB from *V. porci* and *L. lactis* (PDB: 4WD9).

Green is LanB from *V. porci* and cyan is LanB from *L. lactis*. Highlighted are conserved residues Arg83 and Arg14 to portray functional amino acids are conserved despite low overall amino acid identity. Highlighted in red are residues from *V. porci* and yellow are residues in *L. lactis*. Arg464 is also conserved and shown in the bottom left quadrant of the image. Asp121 is conserved and shown in the bottom right quadrant. Asp299 is shown in the top left quadrant. Threonine as position 89 in the N-terminal end of the protein is shown in the top right quadrant (Garg, Salazar-Ocampo and Van Der Donk, 2013; Khusainov *et al.*, 2015; Lagedroste *et al.*, 2020).

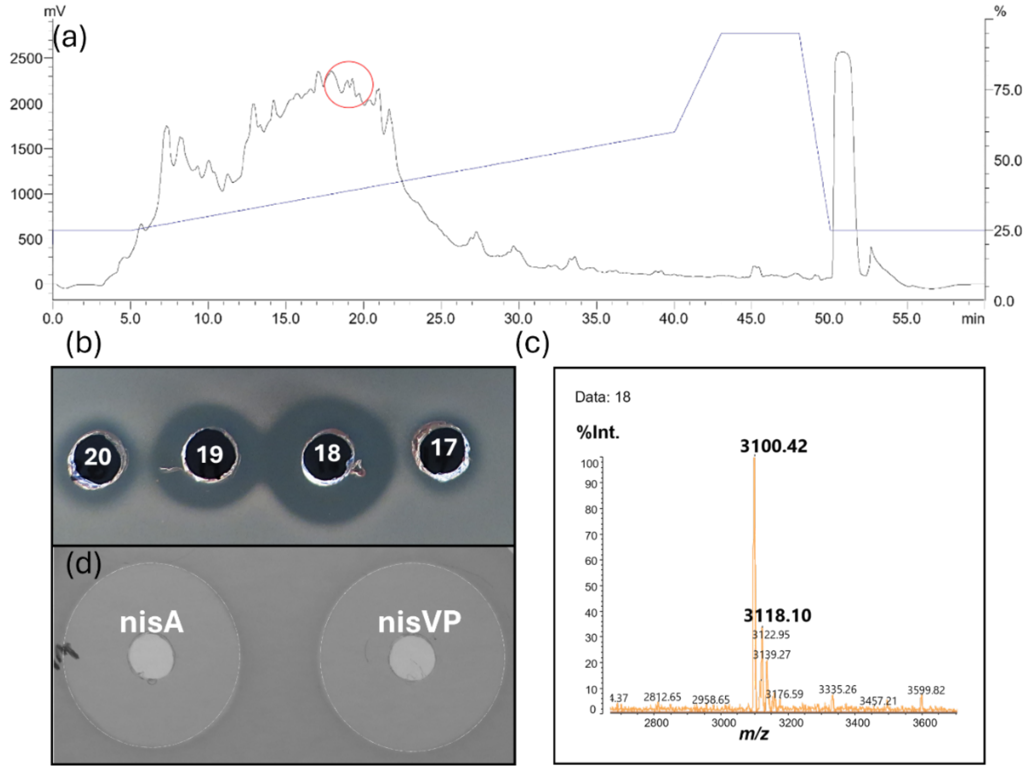

Figure S4. Expression of Nisin VP in *L. lactis* NZ9800. (a) Chromatogram from HPLC of heterologously expressed Nisin VP. The peptide eluted in fractions 18 and 19. **(b)** Well diffusion assay showing antimicrobial activity in fractions 18 and 19. **(c)** Mass spectrometry for the active fraction 18 shows a 3,100 Da peptide as well as peptides missing 1 dehydration (3118Da). A peptide mass corresponding to 2 missed dehydrations (3136Da) was observed in fraction 19 (data not shown). **(d)** Nisin VP is active against *L. bulgaricus* LMG6901 at 30 μM and exhibits a similar zone size as nisin A.

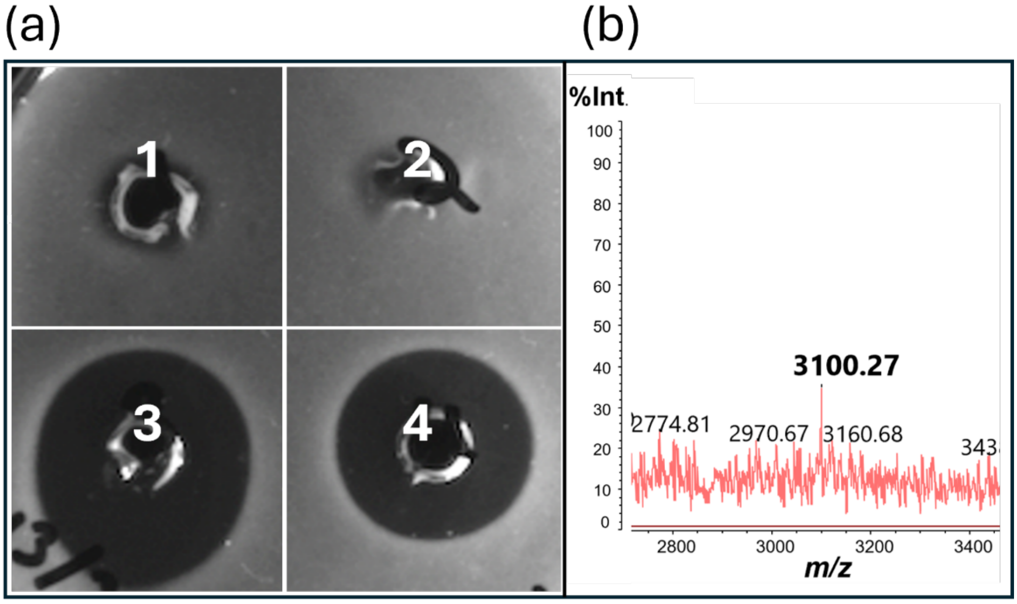

Fig S5. (a) Well Diffusion Assays (WDA) showing induction of nisin VP. Wells 1-2 were inoculated with cell-free supernatant (CFS) from single colonies selected from a non-Nisaplin® containing plate while wells 3-4 were inoculated with CFS from cultures selected from a Nisaplin+ plate and (b) colony mass spec for *Velocimicrobium porci* DSM 107250. The colony mass spec shows a 3100.27 Da peptide produced by the bacterium that corresponds to nisin VP.

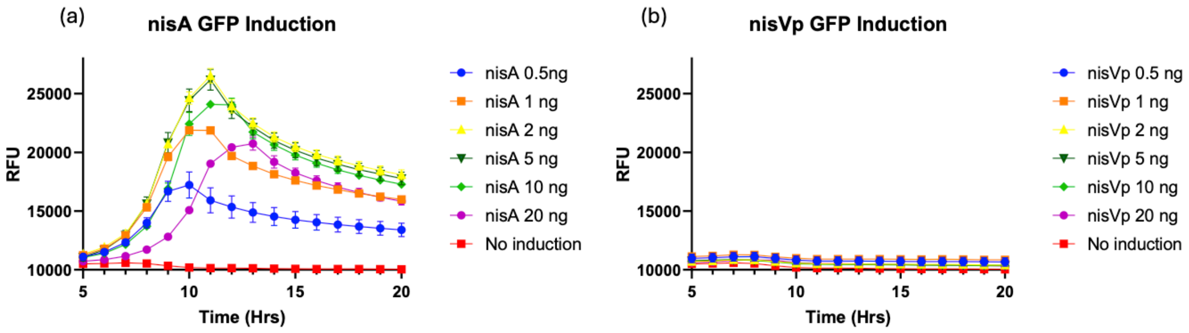

Fig. S6 GFP reporter assay for Nisin A and Nisin VP induction.

(a) Induction capacity of nisin A as determined by expression of GFP under the control of the P*nisA* promoter in *L. lactis* NZ9000 pNZ8150*gfp*+ induced at concentrations between 0.5 ng/ml to 20 ng/ml. Intensity of fluorescence is measured in relative fluorescence units (RFU) **(b)** Nisin VP was unable to induce the NisRK system at comparable concentrations.

Table S10. MIC table for bacitracin and Nisin A for the Nisin A machinery NisI, NisFEG and NisIFEG. NisIFEG was unable to confer bacitracin tolerance to *L. lactis* MG1614.

	**Bacitracin µg/ml**	**Immunity/Resistance conferred**	**Nisin A µM (µg/ml)**	**Immunity/Resistance conferred**
***L. lactis* MG1614**	0.78	n/a	0.0156 (0.052)	n/a
**MG1614 pNZ-nisI**	0.78	0	0.937 (3.125)	60X
**MG1614 pNZ-nisFEG**	0.78	0	1.875 (6.25)	120X
**MG1614 pNZ-nisIFEG**	0.78	0	0.937 (3.125)	60X
**MG1614 pNZ-nsrFP**	3.12	4X	1.875 (6.25)	120X

Table S11. Table of amino acid sequences of nisin-like peptides and the source.

Name	Sequence	Taxonomy	Source
Blauticin	ITSKSLCTPGCVTGILMTCPVQTATCGCQITGK	*Blautia producta*	GCA_002221555.2
Blauticin*	TSKSLCTPGCVTGILMTCAINTATCGCQITGK	*Anaerobutyricum hallii*	MGYG000281903
Nisin G	ITSYSLCTPGCKTGVLMACHLKTATCNCSIIVSK	*S. salivarius DPC6487*	N/A
Nisin G*	ITSYSLCTPGCKTGVLMACHLKTATCNCGFIISK	*Erysipelatoclostridium ramosum*	MGYG000259859
Ce02	VTSVSLCTPGCITGVIMTCTIKTATCGCHVAGK	*Clostridium* sp. E02	GCA_003833015.1
RL8*	ITSKSLCTPGCITGILQTCAIKTATCGCHITGK	*Lacrimispora sp.*	MGYG000004680
VP	ITSKSLCTPGCVTGILQTCAIQTATCGCSITGK	*Velocimicrobium porci* WCA-693-APC-MOT-I	GCA_009696045.1
Moraviensicin	ITSKSLCTPGCVTGVLMGCALKTITCNCSVGIGKK	*Enterococcus moraviensis* ATCC BAA-383	GCA_000394015.1
Novac	VTSVSLCTPGCVTGALQTCPAQTATCGCPISK	*Nocardia vaccinia* NBRC 15922	GCA_001613305.1

Table S12. Primers used for cloning of nisin-like peptides.

Peptide	Primer Name	Sequence
**ALL**	For Primer EcoR1	5’- CATAGAATTCTAGTCTTATAACTATACTGACAATA - 3’
**Blauticin**	BlauRevXba1	5’- AGTTTCTAGATTATTTACCAGTGATTTGACAACCA 3’
**Blauticin Variant (Bvar)**	BlauRevXba1	5’- AGTTTCTAGATTATTTACCAGTGATTTGACAACCA 3’
**Nisin G**	NisGRevXba1	5’- AGTTTCTAGATTATTTTGAAACGATGATTGAACAG
**NisinGvariant**	NisGRevXba1	5’- AGTTTCTAGATTATTTTGAAACGATGATTGAACAG
**NisNovac**	NisNovacRevXba1	5’- AGTTTCTAGATTATTTTGAGATTGGACAACCACA 3’
**NisVelocipor (Vp)**	NisVelociporRevXba1	5’ AGTTTCTAGATTATTTACCAGTGATTGAACAACCA 3’
**CE-02**	NisCE-02RevXba1	5’- AGTTTCTAGATTATTTACCAGCAACGTGACAACCA 3’

**RL8VarMGYG**	RL8VarRevXba1	5’- AGTTTCTAGATTATTTACCAGTGATGTGACAACCA 3’
**Moraviensicin**	MoraRevXba1	5’- AGTT TCTAGA TTATTTTTTACCGATACCAACT 3’
	**pCI372 For**	5′-CGGGAAGCTAGAGTAAGTAG-3′
	**pCI372 Rev**	5′-ACCTCTCGGTTATGAGTTAG-3′
